# RNA-Seq Profiling Identifies Temperature-Responsive Genes in Germinating Urediniospores of *Puccinia striiformis* f. sp. *tritici* Race CYR34-8

**DOI:** 10.3390/jof12080582

**Published:** 2026-08-07

**Authors:** Fei Tao, Hong Han, Hong Tao, Yiping Zou, Xinke Kong

**Affiliations:** 1 College of Plant Protection, Gansu Agricultural University, Lanzhou 730070, China; 2 Academy of Agricultural Sciences of Linxia Hui Autonomous Prefecture, Linxia 731100, China; hanhong5325@163.com; 3 Forest Seedling Service Station of Linxia Hui Autonomous Prefecture, Linxia 731100, China; taohong045@163.com; 4 College of Life Sciences, Shandong Normal University, Jinan 250014, China; zouyiping@sdnu.edu.cn; 5 School of Foreign Languages and Literatures, Lanzhou University, Lanzhou 730070, China; kongxk@lzu.edu.cn

**Keywords:** wheat (*Triticum aestivum* L.), *Puccinia striiformis* f. sp. *tritici*, urediniospore germination, temperature sensitivity, thermal adaptation, RNA-Seq

## Abstract

Wheat stripe rust (yellow rust), a devastating disease caused by the phytopathogen *Puccinia striiformis* f. sp. *tritici* (*Pst*), poses a major threat to global wheat (*Triticum aestivum* L.) production. Under ongoing climate warming, the highly virulent *Pst* race CYR34 has become predominant in China and exhibits increased tolerance to elevated temperatures. Although temperature is known to influence *Pst* urediniospore germination, the molecular mechanisms underlying temperature sensitivity during this process remain poorly understood. In this study, we combined histological examinations with transcriptome sequencing across multiple temperature regimes to identify temperature-responsive genes and characterize the predicted protein association networks in *Pst*. Urediniospore germination of three *Pst* races was tested; stronger heat tolerance was observed in CYR34-8 with 67.40–77.40% germination at 9–16 °C. RNA-Seq analysis identified 89 differentially expressed genes (DEGs) associated with temperature sensitivity, and their expression patterns were validated by qRT-PCR. The DEGs were significantly enriched in ubiquinone and other terpenoid-quinone biosynthesis, glycan degradation, and longevity-regulating pathways. Among these DEGs, genes annotated as encoding an ABC transporter, malate dehydrogenase, Hsp70, and a class 3 lipase were identified as putative hub genes in the predicted protein association network and may be associated with the coordination of temperature responses and metabolic processes.

## 1. Introduction

Stripe (yellow) rust, caused by *Puccinia striiformis* f. sp. *tritici* (*Pst*), is a widely distributed and rapidly spreading airborne disease that severely affects global wheat production [1,2]. The prevalence of stripe rust is strongly dependent on cool and humid climatic conditions, occurring across all major wheat-producing continents [3]. Recently, stripe rust has become increasingly prevalent in the northwestern United States, Australia, India, and China [4,5,6,7]. *Pst* primarily completes its annual infection cycle on wheat during the asexual stage as urediniospores [8]. Although fungicides can effectively control the disease, their extensive use not only leads to environmental degradation but also accelerates pathogen virulence variation and the development of fungicide resistance in *Pst* [9,10]. Extensive field evidence indicates that breeding wheat cultivars with durable resistance is a long-term and effective strategy for managing stripe rust [10]. However, following the emergence of new *Pst* races with high virulence, resistant wheat varieties often lose their resistance within 2–5 years, becoming susceptible varieties and leading to severe and irreversible yield losses [11].

Global climate change has driven frequent genetic variations among phytopathogens, facilitating their adaptation to elevated temperatures [12]. Multiple field surveys have confirmed the emergence and spread of novel, highly virulent *Pst* races that have colonized new regions and have exhibited greater high-temperature tolerance than traditional isolates [13,14]. Previous studies indicate that 13–16 °C represents the optimal temperature range for stripe rust development, which has been proposed as a limiting factor for its spread between the United States and Canada [15]. However, *Pst* isolates collected after 2000 in the eastern United States have been shown to adapt to a broader temperature range of 12–28 °C [11,16]. The Longnan and Tianshui regions of Gansu Province are recognized as key epidemic and transmission centers of stripe rust in China [17,18]. Studies in these regions have shown that the overwintering altitude of *Pst* has increased by approximately 300 m, whereas the oversummering altitude has decreased by 100–300 m. In addition, the average ambient temperature experienced by pathogen populations often exceeds 24 °C, suggesting enhanced adaptation and pathogenicity under higher temperature conditions [19]. Since 2016, the emergence frequency of the new race G22-9 (CYR34) has increased rapidly from 0 to 34.85%, making it the dominant race in Gansu Province. This race exhibits greater pathogenicity, a broader virulence spectrum, and higher parasitic fitness compared with the prevailing physiological races CYR32 and CYR33 [20,21].

As an obligate biotrophic fungus, *Pst* produces a series of specialized infection structures during wheat colonization, including germ tubes, substomatal vesicles, primary hyphae, secondary hyphae, haustorial mother cells and mature haustoria, which develop in a fixed sequential order [22]. Urediniospore germination and germ tube growth are critical prerequisites for successful *Pst* infection [23,24]. Urediniospores also serve as the primary overwintering form of *Pst* and remain metabolically active during winter [25]. As early as 1981, North American researchers developed a stripe rust prediction model using 7 °C as the base temperature for calculating the effective accumulated temperature required for urediniospore germination and infection [26,27]. Temperature not only influences urediniospore germination and infection but is also closely associated with *Pst* variation. After germination and germ tube formation, cytoplasmic connections can form between different *Pst* races, enabling protoplasmic and molecular exchange—a key mechanism contributing to the emergence of new virulent races [28]. Additional studies in mountainous areas around Tianshui have shown that many local *Pst* races exhibit urediniospore multinucleation rates exceeding 1%, which may contribute to changes in pathogen pathogenicity [29,30].

Exploring how temperature modulates *Pst* pathogenicity and genetic variation is essential for understanding stripe rust epidemiology under climate change [31]. The rapid development of high-throughput sequencing technologies has greatly advanced genomic and transcriptomic research on this obligate biotrophic fungus, and whole-genome sequences of multiple *Pst* physiological races have been made available [32,33]. Since 2007, when Peng et al. first constructed a cDNA library from urediniospores of Pst-70, a physiological race of *Pst*, transcriptomic approaches have been widely used to investigate urediniospore germination and infection processes [34,35,36]. Most current transcriptomic studies on *Pst* have focused on effector proteins and single nucleotide polymorphisms (SNPs) associated with pathogen–host interactions [37,38,39,40]. As the primary climatic factor influencing *Pst* overwintering, oversummering, and epidemic dynamics, temperature plays a central role in determining the global distribution and prevalence of stripe rust [3]. However, the molecular regulatory mechanisms underlying *Pst* urediniospore germination under temperature stress remain poorly understood. In this study, we aimed to identify co-expressed genes and characterize their regulatory networks associated with temperature sensitivity during *Pst* urediniospore germination. We found that thermal treatments at 9–16 °C applied for different durations activated thermotolerance-related molecular pathways in CYR34. A set of temperature-responsive DEGs was identified, providing insights into the molecular basis of temperature sensitivity in *Pst*.

## 2. Materials and Methods

### 2.1. Plant Materials, Fungus, and Urediniospore Propagation

The susceptible wheat cultivar Mingxian 169 (MX169) and three dominant Chinese *Pst* races (CYR32-11, CYR33-209, CYR34-8) were provided by the Institute of Plant Protection, Gansu Academy of Agricultural Sciences (Lanzhou, China). To obtain fresh urediniospores, we inoculated MX169 seedlings with the three *Pst* races. Ten to fifteen wheat seeds were sown in 10 × 10 × 10 cm plastic pots, with an inter-seed distance of approximately 1.5 cm. Urediniospores were mixed with sterile water at a volume ratio of 1:6–9, and the mixture was gently stirred with an inoculation needle. The spore suspension containing floating urediniospores was used for seedling inoculation. After inoculation, seedlings were transferred to an artificial climate chamber and cultured at 15 ± 1 °C, 8000–12,000 Lx light intensity and 70–85% relative humidity. Fresh urediniospores were harvested 10–12 days post inoculation and stored at −80 °C for subsequent experiments.

### 2.2. Temperature Treatments and Histopathological Observations

A 10-μm nylon membrane was placed on water-saturated filter paper and used as a support for urediniospore germination [30]. The urediniospores of CYR32-11, CYR33-209 and CYR34-8 race were placed on Nylon membrane and incubated for 6 h, 10 h and 14 h at 9 °C, 12 °C, 14 °C and 16 °C respectively, and the germination of urediniospores was observed under the microscope. The criterion for germination was that the length of the germ tube of the urediniospores was greater than or equal to the radius of the urediniospores when examined microscopically. The urediniospores germination rate was calculated by randomly observing three visual fields under a microscope with a 20×. The germination rate of urediniospore were evaluated using the following model: $y=\frac{a}{b}$ × 100%, where y is the germination rate of urediniospore, a is the number of germinated urediniospores, b is the total urediniospores [30].

### 2.3. RNA-Seq

Only urediniospores of *Puccinia striiformis* f. sp. *tritici* race CYR34-8 were used for transcriptome sequencing. The experiment followed a factorial design comprising four incubation temperatures (9, 12, 14, and 16 °C), three incubation times (6, 10, and 14 h), and three independent biological replicates for each temperature–time combination, resulting in a total of 36 samples (4 temperatures × 3 time points × 3 biological replicates). The four temperature treatments were performed separately. Under each temperature condition, germinating urediniospores were collected independently at 6, 10, and 14 h. Samples collected at different temperatures and incubation times were processed separately and were not pooled.

The three biological replicates were generated through three independent inoculation, pathogen propagation, and urediniospore-harvesting experiments. For each biological replicate, CYR34-8 was independently inoculated onto a separate batch of susceptible wheat seedlings, propagated separately, and harvested independently. The urediniospores obtained from each independent harvest were subsequently subjected to the four temperature treatments and sampling procedures. Thus, the biological replicates did not represent subdivisions of a single urediniospore harvest.

Immediately after collection, each sample was snap-frozen in liquid nitrogen and stored at −80 °C until RNA extraction. Total RNA was purified via the TRIzol method (Thermo Fisher Scientific, Waltham, MA, USA), and residual genomic DNA was digested with 1 U/g DNase I (Thermo Fisher Scientific, Waltham, MA, USA). RNA integrity and concentration were evaluated on an Agilent 2100 Bioanalyzer (Agilent Technologies, Waldbronn, Germany). The RNA samples were submitted together to minimize potential technical batch effects associated with separate sample submissions, library preparation, and sequencing runs. Thirty-six samples were used to construct paired-end (PE) cDNA libraries, which were sequenced on the Illumina HiSeq-2500 platform by BMKGENE (Beijing, China). All sequencing data met strict quality criteria: each library yielded over 4 Gb of raw reads. All libraries satisfied strict sequencing quality standards with an average Q30 ratio above 90% (Table S2). Raw sequence data were preprocessed with Trimmomatic (v.0.4) to remove adapter sequences and low-quality bases [41]. Processed clean reads were mapped to the public reference genome of *Puccinia striiformis* f. sp. *tritici* genome (https://ftp.ncbi.nlm.nih.gov/genomes/all/GCA/001/191/645/GCA_001191645.1_P_striiformis_V1/GCA_001191645.1_P_striiformis_V1_genomic.gff.gz accessed on 15 April 2025) using TopHat2 (v2.1.0). Subsequent transcript assembly was completed using Cufflinks (v2.2.1) and Cuffmerge (v2.2.1) [41]. The DEG analyses were evaluated using software Cuffdiff (v 2.2.1). Genes with *padj* < 0.05 and |log_2_ FC| > 2 were regarded as temperature-responsive DEGs [42].

### 2.4. Functional Annotation and Enrichment

Transcript functional annotation was conducted using BLASTx (v2.14.0) with an (*E*-value < 1 × 10^−5^) against KEGG, Nr, KOG and Swiss-Prot databases. Gene Ontology (GO) annotations were obtained using the Blast2GO (v6.0) program. We performed enrichment analysis for GO terms and KEGG pathways, and items with *padj* < 0.05 were considered statistically significant [43].

### 2.5. Genomic Localization of DEGs

Identified DEGs were mapped to the *Pst* genome. These mapped DEGs were adopted as query sequences for use with BLASTN (with an *E*-value < 1 × 10^−50^) against the predicted mRNA database of the *Pst* genome to search location information on chromosomes [44].

### 2.6. Protein–Protein Interaction (PPI) Network

Proteins encoded by identified DEGs were used to predict protein–protein interactions (PPIs) using the STRING database (*E*-value < 1 × 10^−10^, http://string-db.org/ 15 July 2026). PPIs with combined confidence scores greater than 0.7 were selected [45] and visualized with CYTOSCAPE (v.2.8, http://cytoscape.org/ 15 July 2026) [46].

### 2.7. RNA-Seq Data Submission

The raw data have been submitted to the NCBI Sequence Read Archive (SRA) database under accession numbers from SRR34732539 to SRR34732574 (Supplementary File S1).

### 2.8. Quantitative Reverse Transcription PCR (qRT-PCR)

Thirteen representative DEGs were chosen for qRT-PCR validation (Table S1). UltraSYBR Mixture (Kangwei, Beijing, China) and iQ^TM^ 5 (Bio-Rad, Hercules, CA, USA) were used for qRT-PCR analysis of all reactions according to the manufacturer’s instructions. Data were collected from three replicate experiments—the samples used for qRT-PCR were the same as those used for RNA-Seq, each consisting of at least three technical repeats. Negative controls that lacked templates were also included. Housekeeping genes *EF-1α* and *Act* from *Pst* served as reference genes for expression normalization. The expression ratio of each gene was calculated by using the relative expression software tool of REST (v2.0.13) [47].

## 3. Results

### 3.1. Histopathological Observation of Urediniospore Germination Under Different Temperature Treatments

**Figure 1:**
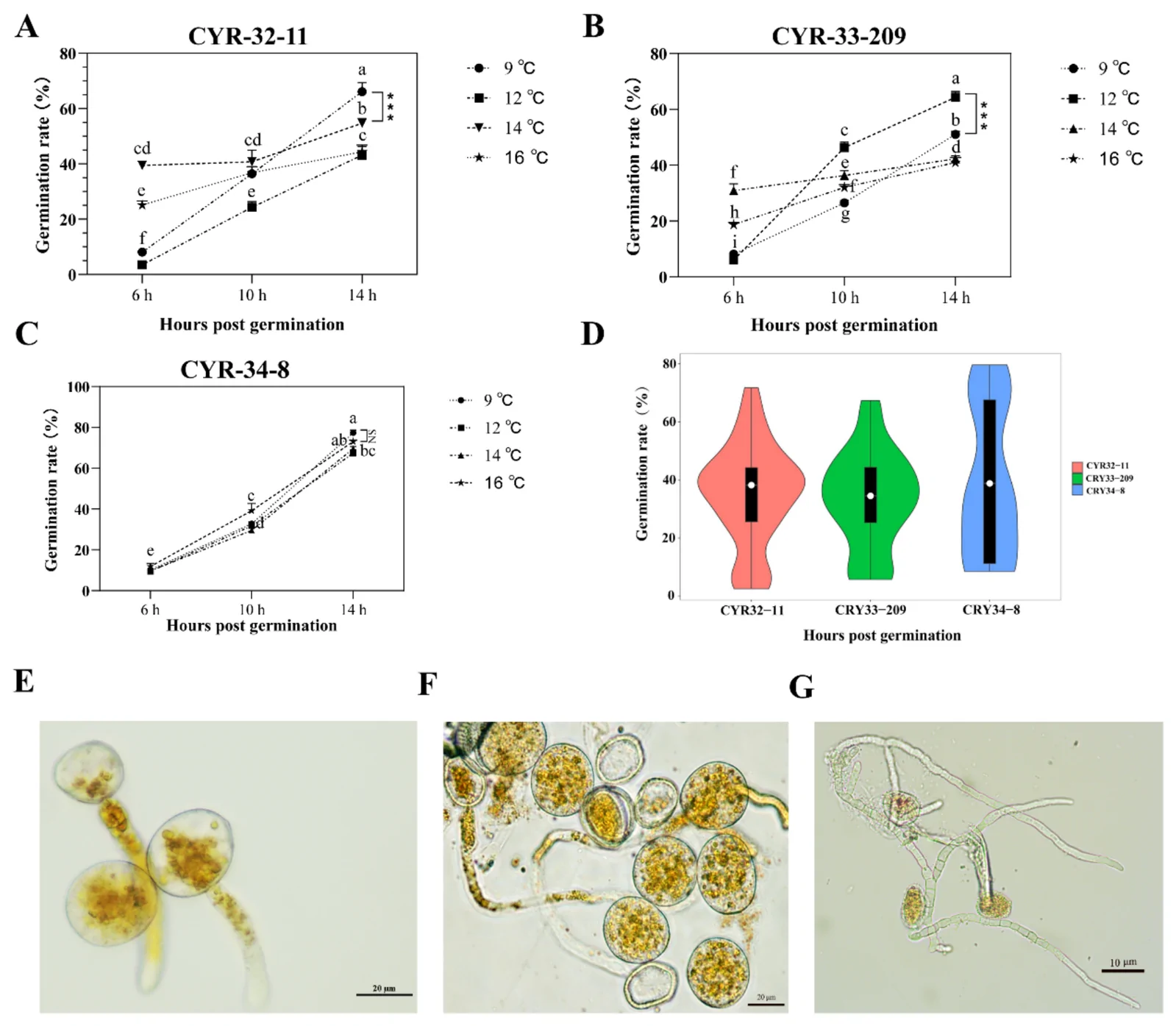
Temperature-dependent urediniospore germination in three *Puccinia striiformis* f. sp. *tritici* races. (**A**) Germination rate of CYR32-11 at different temperatures. (**B**) Germination rate of CYR33-209 at different temperatures. (**C**) Germination rate of CYR34-8 at different temperatures. (**D**) Comparison of germination rates among the three races under different temperatures. (**E**–**G**) Representative micrographs of urediniospore germination at 6, 10, and 14 h, respectively. Scale bars are indicated in each panel. Different lowercase letters (a–h) indicate statistically significant differences between groups (*p* < 0.05). Asterisks (*) denote significant differences for pairwise comparisons.

The germination of urediniospores from three prevalent *Puccinia striiformis* f. sp. *tritici* races in China, namely CYR32-11, CYR33-209, and CYR34-8, was evaluated under different temperature conditions. After 14 h of incubation, CYR32-11 showed the highest germination percentage at 9 °C (66.10%), followed by 14 °C (54.73%) (Figure 1A). For CYR33-209, the maximum germination percentage was obtained at 12 °C (64.33%), which was significantly higher than that at 9 °C (*p* < 0.05) (Figure 1B). In contrast, CYR34-8 maintained consistently high germination percentages across 9 °C, 12 °C, 14 °C, and 16 °C, ranging from 67.40% to 77.40%, with no significant differences among temperatures (Figure 1C). Comparative analysis showed that CYR34-8 exhibited a higher overall germination capacity than the other two races across all tested temperatures (Figure 1D). Microscopic observations showed that CYR34-8 urediniospores formed germ tubes within 6 h at 16 °C (Figure 1E), which became markedly elongated by 10 h (Figure 1F) and exhibited germ tube anastomosis at 14 h (Figure 1G).

### 3.2. Transcriptional Responses of Puccinia striiformis *f. sp.* tritici CYR34-8 to Gradient Temperatures

**Figure 2:**
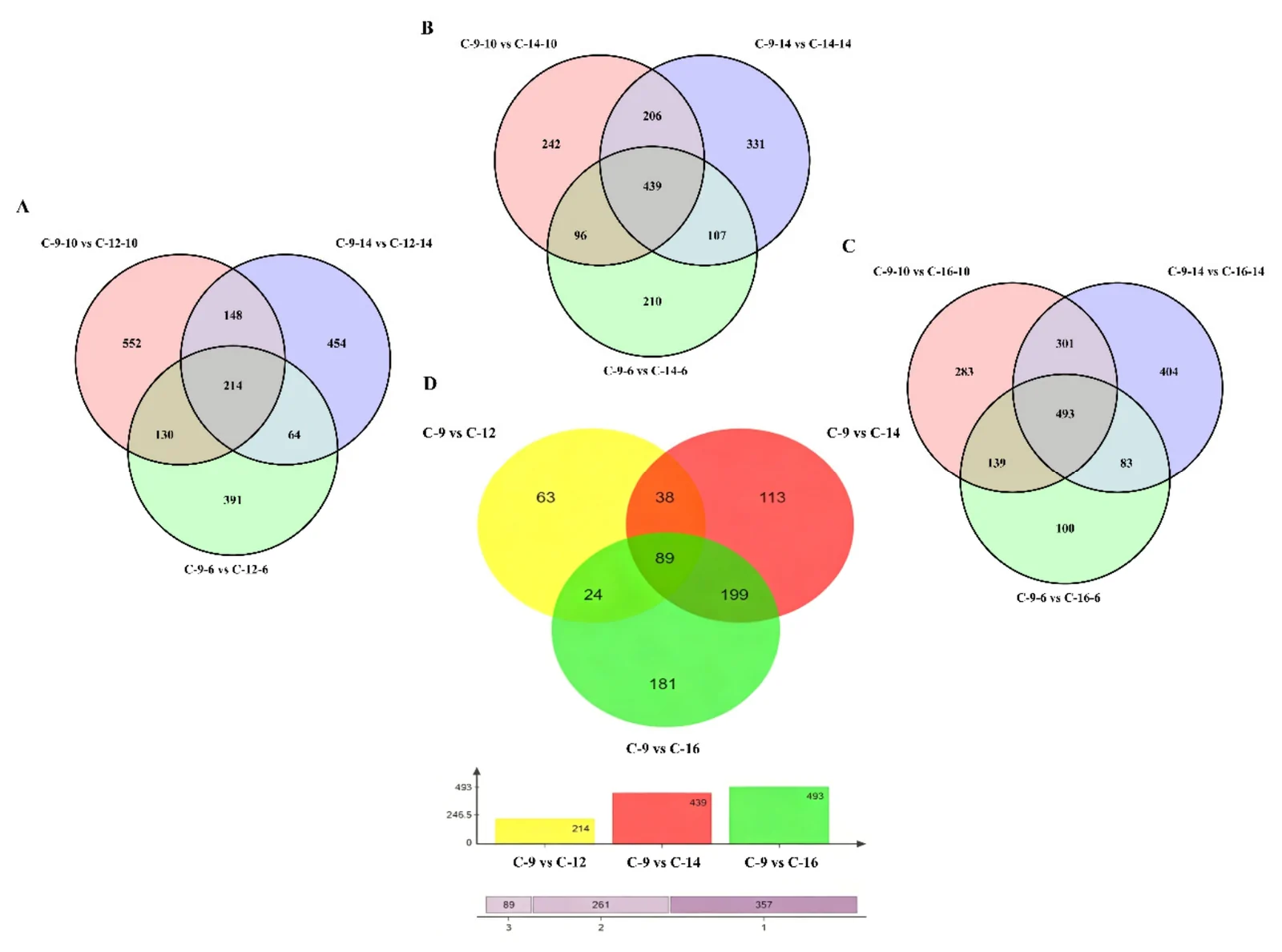
Distribution of differentially expressed genes (DEGs) under different temperature treatments. (**A**) Distribution of DEGs in the 9 °C vs. 12 °C comparison. (**B**) Distribution of DEGs in the 9 °C vs. 14 °C comparison. (**C**) Distribution of DEGs in the 9 °C vs. 16 °C comparison. (**D**) Distribution of DEGs in the 12 °C, 14 °C and 16 °C groups relative to the 9 °C control.

Principal component analysis (PCA) of 36 transcriptome samples from *Puccinia striiformis* f. sp. *tritici* race CYR34-8 confirmed robust consistency among biological replicates of each treatment group. Meanwhile, samples incubated at 14 °C and 16 °C clustered together (Figure S1), verifying that elevated temperature serves as the primary determinant of genome-wide gene expression variation. To further elucidate the molecular mechanisms underlying thermal adaptation in CYR34-8, transcriptome sequencing and differential expression analysis were performed under different temperature treatments (12 °C, 14 °C, and 16 °C), with the 9 °C treatment as the control. A total of 214, 349, and 493 differentially expressed genes (DEGs) were identified in the 12 °C, 14 °C, and 16 °C groups, respectively. The number of DEGs increased with increasing temperature (Figure 2A–C). To identify genes potentially involved in temperature-responsive regulation, an intersection analysis of DEGs from the different temperature comparisons was conducted. A total of 89 DEGs were shared among the three comparisons, which were considered putative key candidates associated with thermosensitive regulation (Figure 2D).

### 3.3. Verification of RNA-Seq Analysis by qRT-PCR

**Figure 3:**
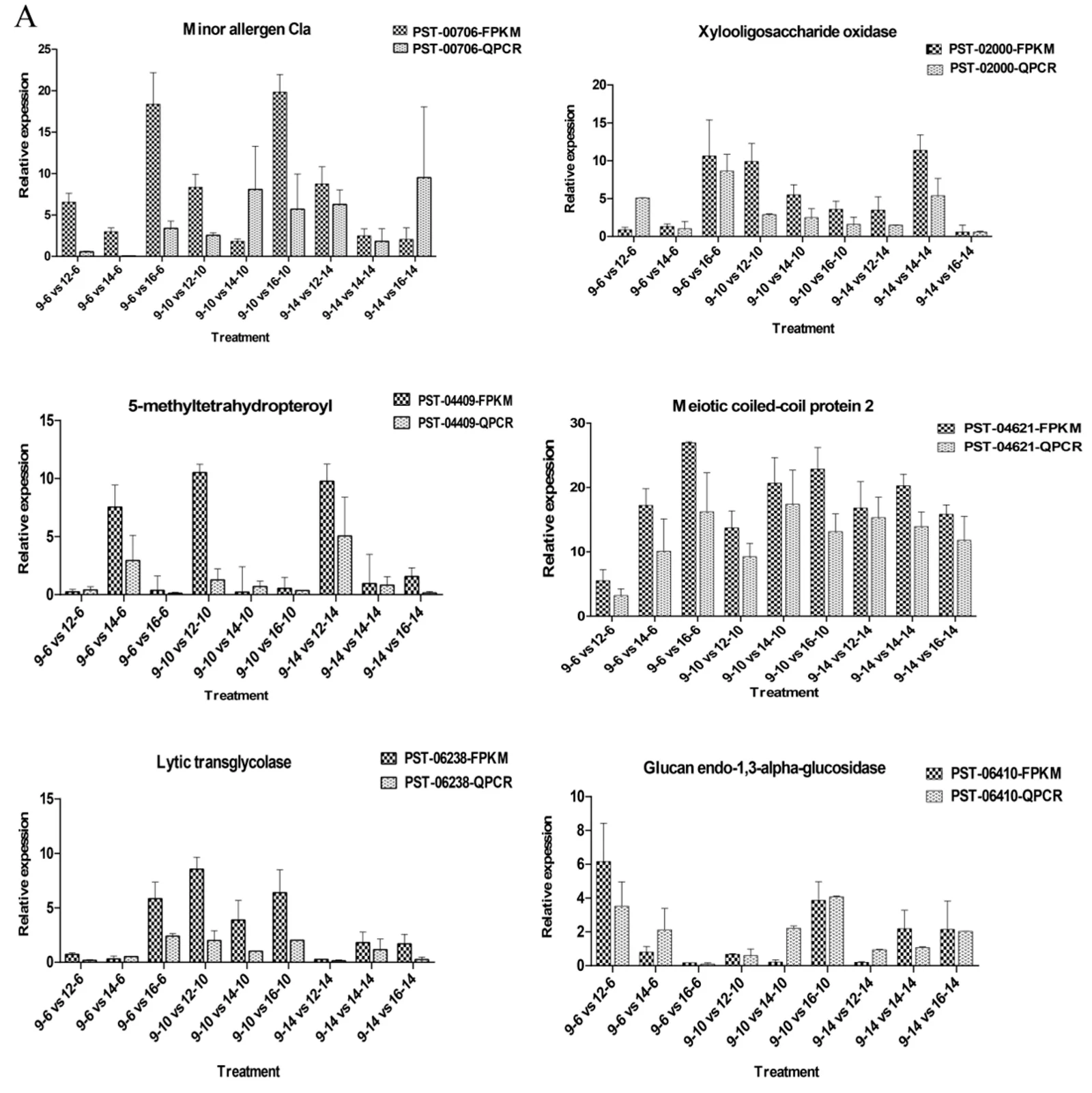

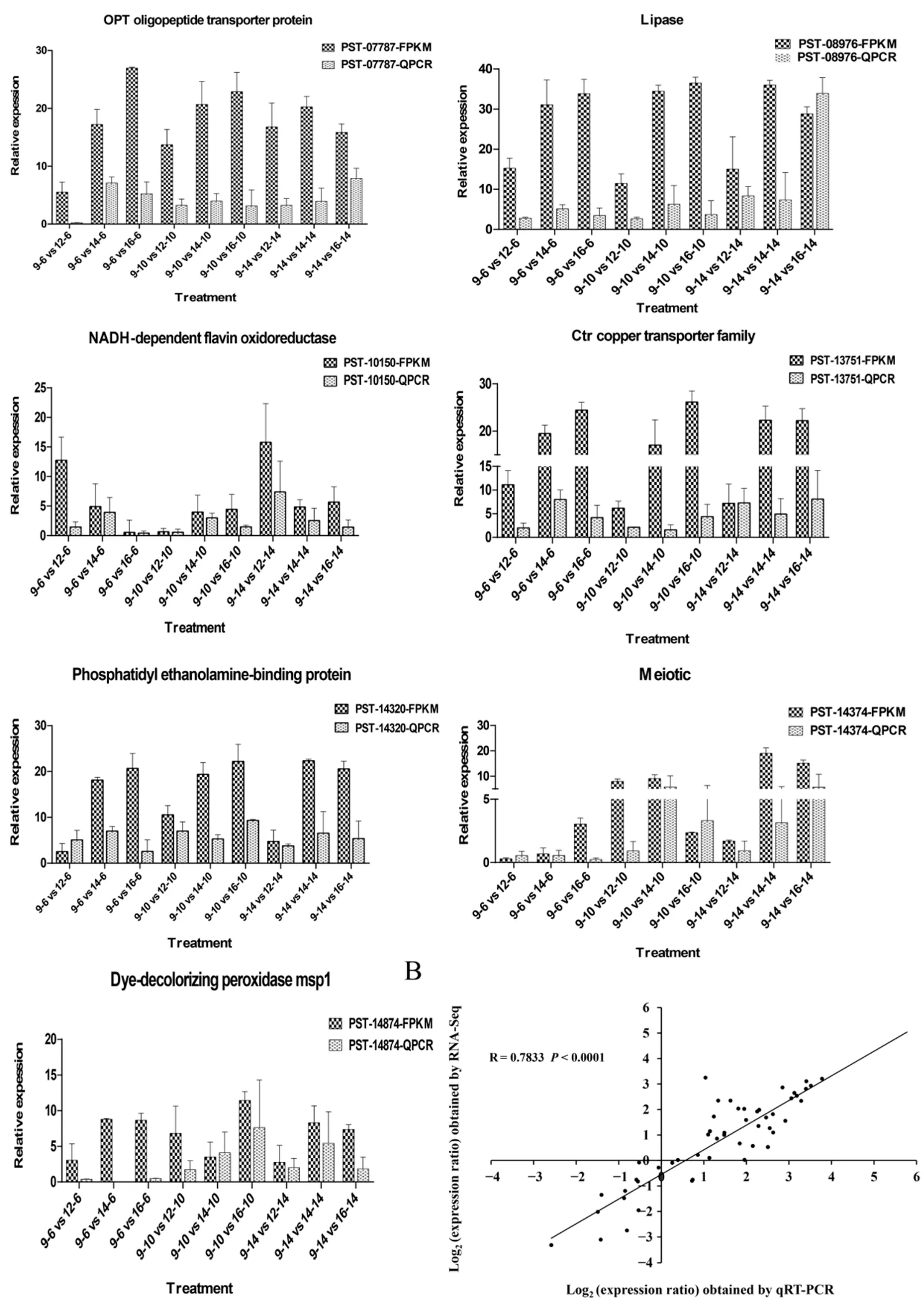
Validation of RNA-Seq results by qRT-PCR. (**A**) Relative expression patterns of 13 selected DEGs determined by qRT-PCR and RNA-Seq. Error bars represent the mean ± SE of three independent biological replicates. (**B**) Correlation between the log_2_ fold changes estimated by RNA-Seq and qRT-PCR. Pearson’s correlation coefficient and the corresponding *p*-value are shown.

To validate the RNA-Seq data, selected DEGs were verified by qRT-PCR. *EF-1α* and *Act* were used as dual reference genes, and samples collected at 9 °C served as the control. Thirteen DEGs with distinct expression patterns were randomly selected for validation (Figure 3A). The expression trends determined by qRT-PCR were generally consistent with those obtained by RNA-Seq. A significant positive correlation was observed between the log_2_ fold changes estimated by RNA-Seq and qRT-PCR (Pearson’s *r* = 0.7833, *p* < 0.05; Figure 3B).

### 3.4. Expression Patterns and Genomic Distribution of Temperature-Responsive DEGs

**Figure 4:**
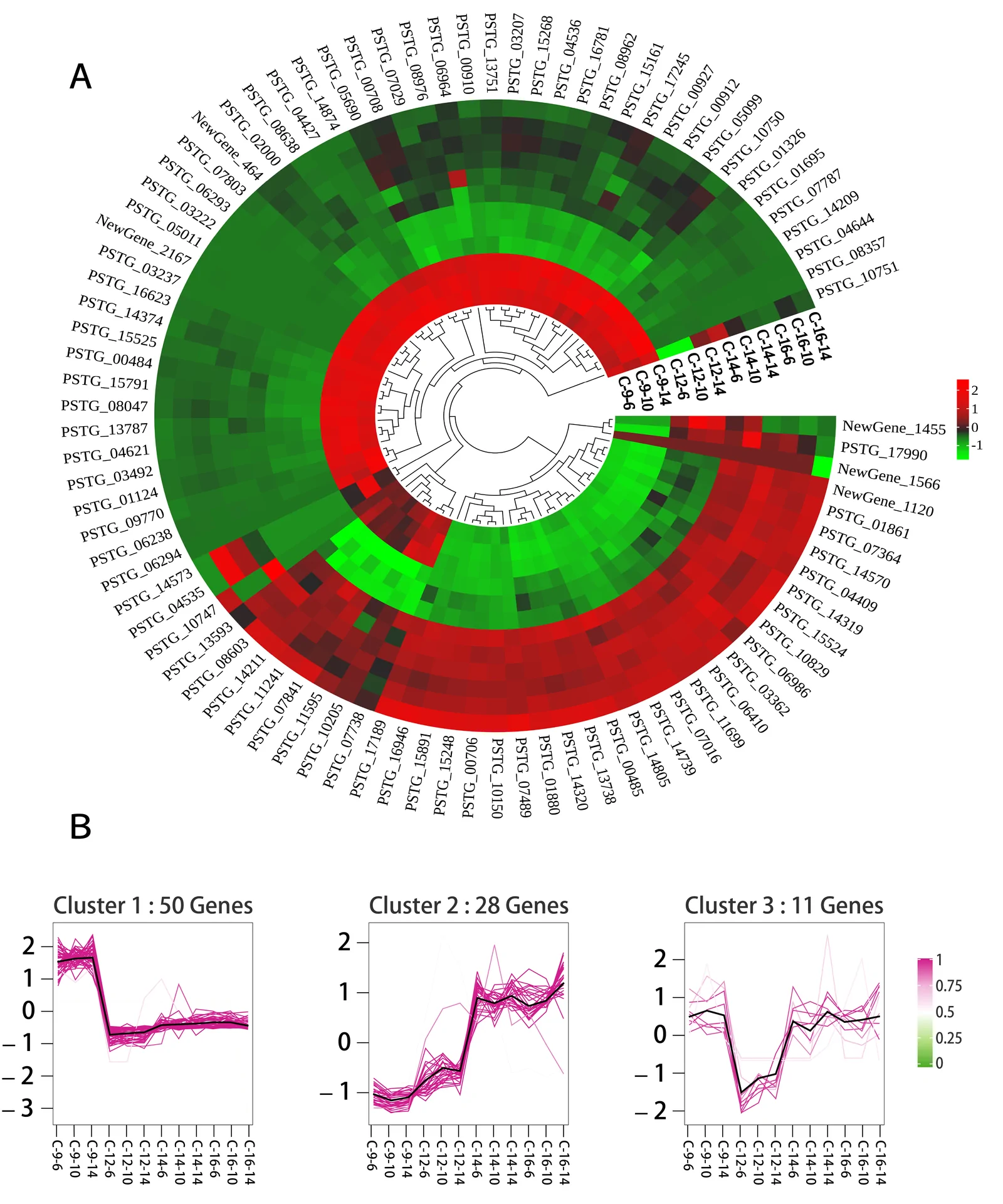
Expression profiles and clustering analysis of DEGs in *Puccinia striiformis* f. sp. *tritici* race CYR34-8 under different temperature treatments. (**A**) Circular heatmap of 89 DEGs. Red indicates upregulated genes, green indicates downregulated genes; inner dendrogram shows gene clustering, outer ring displays gene IDs. (**B**) Three distinct expression clusters of DEGs. Pink lines represent individual gene expression trends, black lines denote the average expression pattern of each cluster (Cluster 1: 50 genes, Cluster 2: 28 genes, Cluster 3: 11 genes).

**Figure 5:**
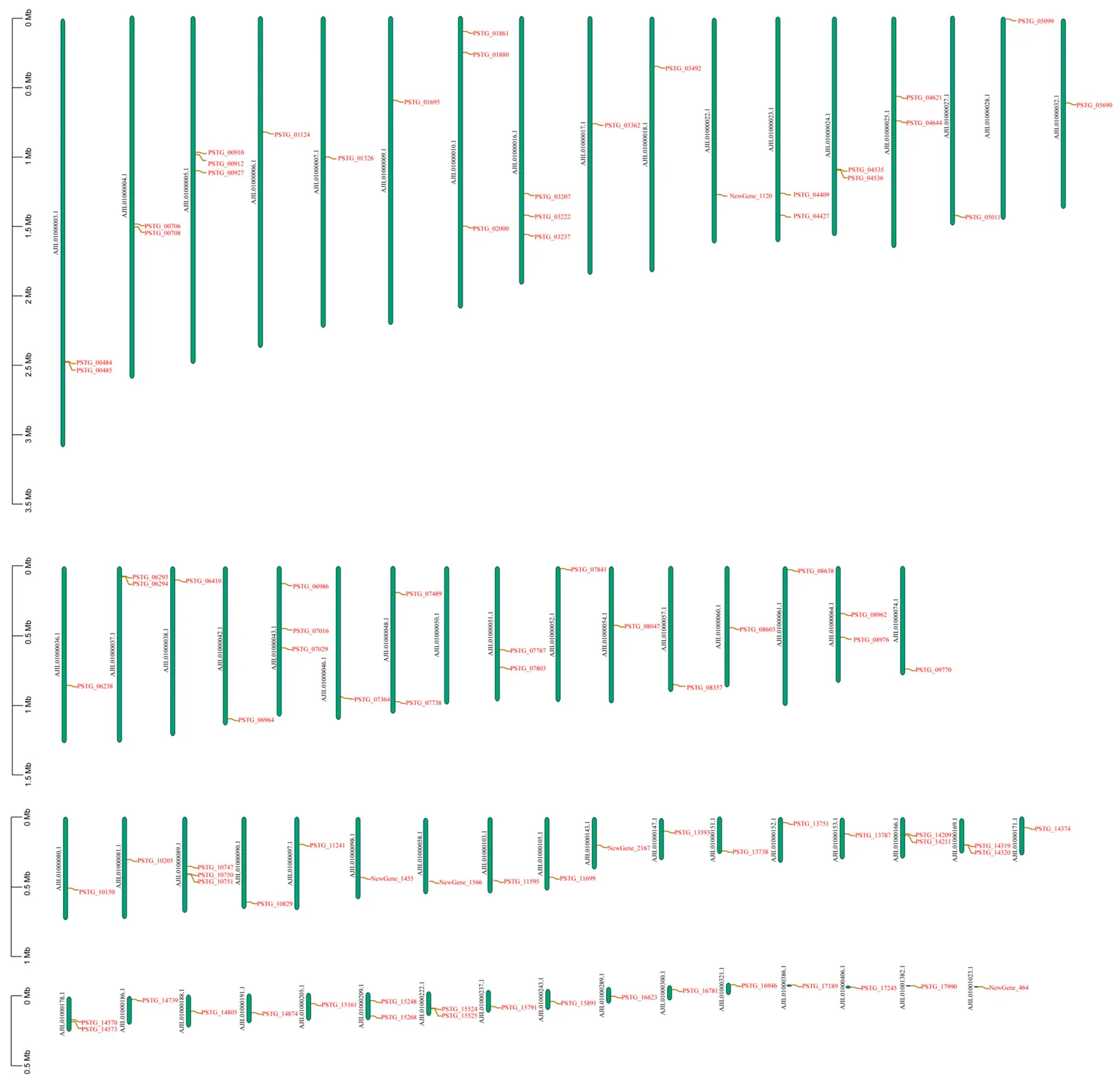
Genomic distribution of 89 temperature-responsive DEGs across the scaffolds of the *Puccinia striiformis* f. sp. *tritici* (CYR34-8) reference genome. Vertical green bars represent genome scaffolds of *Pst*; the left-side scale shows genomic physical distance (Mb). Red labels denote the positions of all 89 DEGs, which are broadly distributed across the genome.

To further investigate the temperature-dependent expression patterns of genes in *Puccinia striiformis* f. sp. *tritici* CYR34-8, DEGs were subjected to heatmap visualization and expression clustering analyses (Supplementary File S2). The heatmap revealed that 55.06% of the DEGs exhibited high expression at 9 °C and were down-regulated at 12 °C, whereas 41.57% were up-regulated at 14–16 °C (Figure 4A). Expression clustering analysis identified three major clusters. Cluster 1 comprised 50 genes whose expression decreased with increasing temperature and peaked at 9 °C, suggesting potential roles in low-temperature adaptation. Cluster 2 contained 28 genes whose expression increased with temperature and reached maximum levels at 14–16 °C, indicating possible involvement in high-temperature responses. Cluster 3 consisted of 11 genes exhibiting fluctuating expression patterns across temperatures, suggesting more complex regulatory mechanisms influenced by temperature and potentially other factors (Figure 4B). Further genomic‑scaffold localization analysis showed that these genes were widely distributed across 66 scaffolds throughout the genome, with no apparent clustering pattern (Figure 5).

### 3.5. Functional Dissection of Core Biological Pathways Enriched in Temperature-Regulated DEGs

To further clarify the functional roles of temperature-responsive differentially expressed genes (DEGs) in *Puccinia striiformis* f. sp. *tritici* race CYR34-8, KEGG pathway enrichment analysis was performed (Table 1). A total of 10 KEGG pathways were significantly enriched (*padj* < 0.05; Table 1), among which ubiquinone and other terpenoid-quinone biosynthesis (ko00130), other glycan degradation (ko00511), and longevity regulating pathway (ko04213) were the most prominent. Specifically, *PSTG_00706* and *PSTG_14805*, which were predicted to encode a minor allergen Cla-like protein and an acyl-CoA ligase, respectively, were annotated to ko00130 pathway. Meanwhile, three DEGs predicted to encode heat shock proteins—*PSTG_06964* (Hsp78), *PSTG_10205* (Hsp70), and *PSTG_13593* (Hsp104)—were annotated to the longevity-regulating pathway (ko04213).

GO enrichment analysis was further conducted to classify the functions of these DEGs (Table S3). In the biological process (BP) category, DEGs were mainly enriched in DNA repair, phosphate ion transport, sulfur compound metabolism, protein maturation, and cell wall disassembly. In the cellular component (CC) category, the most significantly enriched terms were integral component of membrane, intrinsic component of membrane, and membrane. In the molecular function (MF) category, DEGs were predominantly associated with oxidoreductase activity, hydrolase activity, ubiquitin protein ligase activity, and transmembrane transporter activity. GO enrichment profiles exhibited high consistency with KOG annotations (Figure S2).

### 3.6. Predicted Protein Association Network of Temperature-Responsive DEGs and Identification of Putative Hub Genes

**Figure 6:**
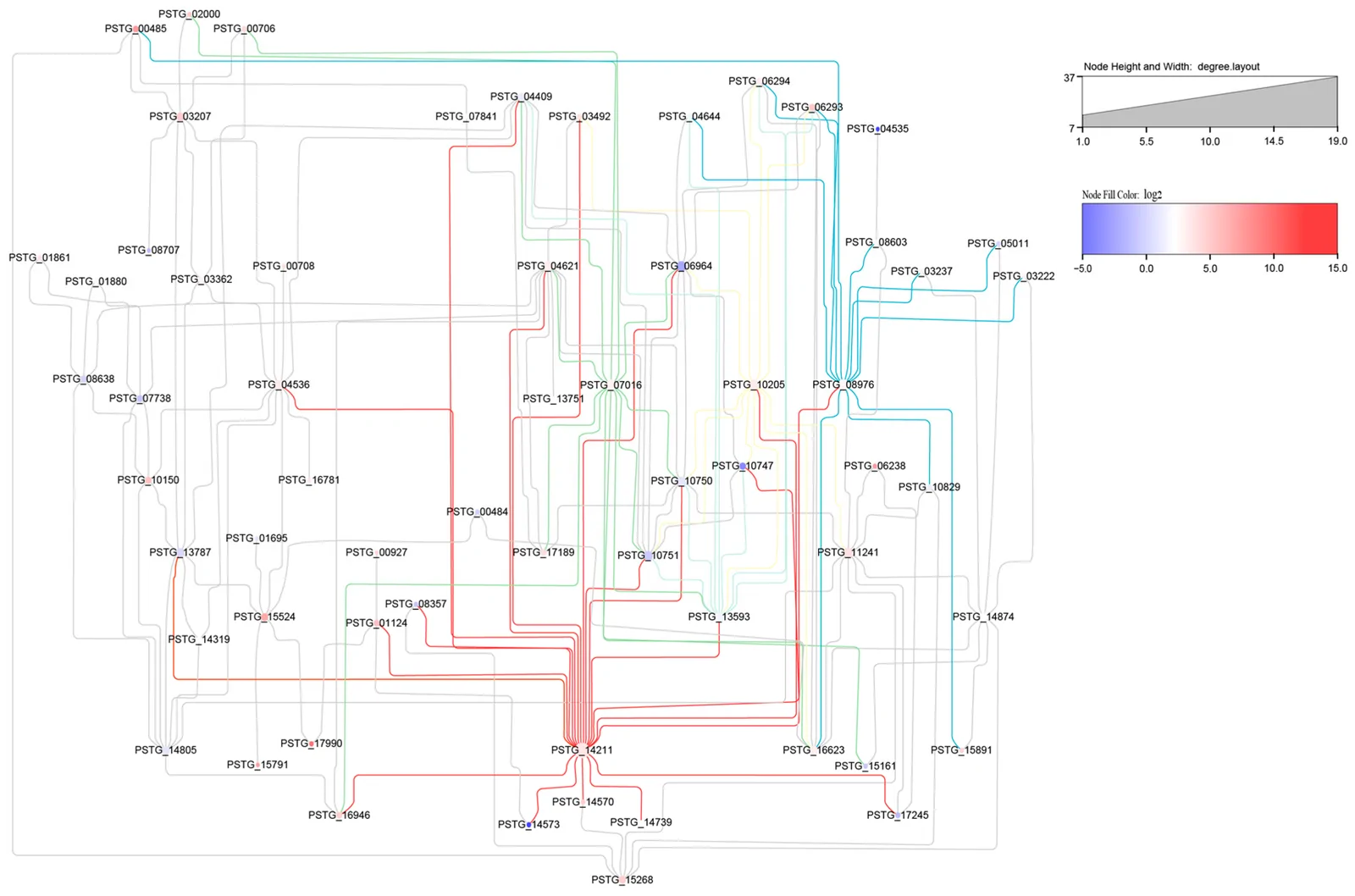
Protein–protein interaction network of 89 DEGs responding to temperature fluctuations. Each node represents an individual DEG, and node color indicates the gene expression level (log_2_^FPKM^). Node size is proportional to the interaction degree. Four key hub genes are highlighted in red, green, blue, and yellow, interacting with 19, 12, 12, and 11 partner genes, respectively. The PPI network was constructed using the STRING database and visualized with CYTOSCAPE software(v.2.8).

A predicted protein association network was constructed for the proteins encoded by the 89 DEGs using the STRING database. Of these, 61 were included in the resulting network based on associations inferred from available homologous proteins and database evidence (Figure 6). Four genes were putatively identified as hubs based on their relatively high node degrees: *PSTG_14211* (red; ABC transporter; log_2_^FPKM^ = 6.21), which was predicted to be involved in transmembrane transport of multiple substrates and is connected to 19 other proteins in the network; *PSTG_07016* (green; malate dehydrogenase; log_2_^FPKM^ = 5.50), which participates in the tricarboxylic acid cycle and central carbon metabolism and is connected to 12 genes; *PSTG_08976* (blue; class 3 lipase; log_2_^FPKM^ = 5.42), which catalyzes lipid hydrolysis and fatty acid metabolism and is connected to 12 genes; and *PSTG_10205* (yellow; Hsp70 protein; log_2_^FPKM^ = 5.94), a core molecular chaperone involved in protein folding and temperature stress adaptation, which connected to 11 genes. These genes were functionally grouped into temperature stress response (heat shock protein family), transmembrane transport, carbohydrate hydrolysis and metabolism, lipid metabolism, central carbon metabolism, and intracellular redox reaction processes. log_2_^FPKM^ values ranged from −4.60 to 12.22, suggesting that the four putative hub genes may act as connectors linking thermal response with diverse metabolic pathways.

## 4. Discussion

As global climate warming continues to reshape the geographical distribution and population structure of phytopathogenic fungi, temperature has become a decisive environmental factor influencing the survival, reproduction, and epidemic dynamics of *Puccinia striiformis* f. sp. *tritici* (*Pst*), the causal agent of wheat stripe rust [3,5,31,48]. Historically, *Pst* was characterized as a cool-adapted pathogen, with urediniospore germination and infection largely restricted at elevated temperatures [1,11]. However, emerging virulent *Pst* races, such as CYR34, have gradually dominated field populations across major wheat-growing regions in China, exhibiting markedly improved thermotolerance compared with traditional races [48,49,50]. Although previous transcriptomic studies of *Pst* have mainly focused on pathogen–host interactions, effector identification, and genetic variation [10,37,40,51], the molecular mechanisms underlying temperature sensitivity during urediniospore germination remain poorly understood. In the present study, we combined histological observations and RNA-Seq to compare thermal adaptability among three representative Chinese yellow rust races and systematically characterize temperature-responsive genes and their regulatory networks in the dominant race CYR34.

Previous studies have demonstrated that temperature is the primary abiotic factor governing urediniospore germination, germ tube growth, and subsequent infection of *Pst* [3,8]. Early biological investigations confirmed that traditional *Pst* races prefer low-temperature environments, with germination capacity declining sharply when ambient temperatures exceed 14 °C [15,27]. However, field monitoring and phenotypic assays have revealed that newly emerged *Pst* races exhibit enhanced tolerance to higher temperatures compared with historical isolates [14]. Consistent with these findings, histological observations of urediniospore germination (Figure 1) revealed obvious inter-racial divergence in temperature preference. The conventional races CYR32-11 and CYR33-209 reached their maximum germination rates at 9 °C and 12 °C, respectively, with the cool-adaptive characteristics of classic *Pst* populations. In contrast, CYR34-8 maintained stable and high germination rates across the entire 9–16 °C temperature range, with no significant differences among treatments. Microscopic observations further confirmed that CYR34-8 completed germ tube formation at 6 h, elongation at 10 h, and germ tube anastomosis at 14 h even at 16 °C. Germ tube anastomosis is known to facilitate heterokaryon formation and genetic recombination in rust fungi, thereby promoting the emergence of new virulent variants and increasing population genetic diversity [28,31]. The urediniospore germination assays in this study indicate that CYR34-8 possesses a broad thermal adaptability, which is consistent with observed field population dynamics [20,21]. Owing to its phenotypic advantages, this race has occupied broader ecological niches and gradually replaced traditional races across China under climate warming [21].

Transcriptional reprogramming is a fundamental strategy by which phytopathogenic fungi perceive and adapt to external temperature stress, and this mechanism has been partially explored in previous transcriptomic studies of *Pst* [40,52]. In line with this consensus, our RNA-Seq analysis revealed that increasing temperature induced pronounced transcriptome-wide changes in gene expression in CYR34 urediniospores. Using 9 °C as the baseline control, we identified 214, 349, and 493 differentially expressed genes (DEGs) at 12 °C, 14 °C, and 16 °C, respectively, demonstrating a progressive increase in the number of DEGs with rising temperature (Figure 2). This positive correlation between temperature elevation and DEG abundance is a conserved stress-response pattern in filamentous fungi, reflecting the gradual enhancement of cellular adaptation to thermal stimuli [50]. Through intersection analysis of the three DEG sets, we identified 89 core temperature-responsive DEGs, which were clustered into three distinct expression profiles corresponding to low-temperature adaptation, high-temperature induction, and complex fluctuating responses across temperature gradients (Figure 4). These DEGs were distributed across 66 scaffolds of the *Pst* genome, indicating that thermal adaptability is a complex quantitative trait governed by multiple dispersed genes rather than a single gene cluster. Collectively, these genes represent key candidate genes associated with temperature responses in CYR34-8.

Functional enrichment analyses based on KEGG (Table 1) and GO (Table S3) databases further revealed that the core temperature-responsive DEGs coordinately mediate the thermal adaptation of CYR34-8 through multiple interconnected biological pathways. Notably, the longevity-regulating pathway was significantly enriched, in which three key heat shock proteins, PSTG_06964 (Hsp78), PSTG_10205 (Hsp70), and PSTG_13593 (Hsp104), were identified. As conserved core components of the cellular protein quality control (PQC) machinery, these heat shock proteins play indispensable roles in maintaining proteostasis under heat stress [53,54]. Studies in model yeast have demonstrated that thermal stress induces the accumulation of misfolded proteins, and the Hsp70-Hsp104 chaperone system is essential for disaggregating stress-induced protein aggregates and restoring cellular function during recovery. The temperature-responsive expression of these putative heat shock protein genes is consistent with the possibility that protein quality-control processes are involved in the response of CYR34-8 to temperature variation. Nevertheless, the current transcriptomic data do not establish that these genes directly stabilize proteins or confer the broader germination-temperature range observed in CYR34-8 [55]. The enrichment of genes annotated to ubiquinone and other terpenoid-quinone biosynthesis raises the possibility that redox-related metabolism is involved in the response to temperature variation. Similarly, the enrichment of other glycan degradation annotations may reflect changes in carbohydrate metabolism or cell-wall-associated processes during germination. These interpretations remain hypotheses because ROS levels, antioxidant capacity, cell wall composition, and the activities of these gene products were not assayed in this study [56,57]. Consistently, GO enrichment analysis associated the DEGs with activation of DNA repair, redox regulation, and ion transport processes, collectively contributing to the maintenance of genomic stability and intracellular homeostasis during temperature fluctuations.

Four proteins predicted to represent an ABC transporter (*PSTG_14211*), malate dehydrogenase (*PSTG_07016*), a class 3 lipase (*PSTG_08976*), and Hsp70 (*PSTG_10205*) displayed relatively high connectivity in the STRING-based association network. They act as central nodes linking thermal stress response with diverse metabolic pathways (Figure 6). ABC transporters are ubiquitous membrane proteins in phytopathogenic fungi and are responsible for transmembrane transport of ions, carbohydrates, and secondary metabolites, and they also participate in intracellular ion homeostasis and detoxification of harmful compounds [58,59]. Malate dehydrogenase, a key enzyme in the tricarboxylic acid cycle that fuels urediniospore germination and germ tube growth, undergoes temperature-dependent conformational changes that help maintain catalytic activity under elevated temperatures [60,61,62]. Class 3 lipases participate in lipid metabolism and modulate cell membrane fluidity, thereby facilitating adaptation to temperature fluctuations [63,64]. As a core molecular chaperone, Hsp70 interacts with a wide range of downstream client proteins and serves as a key regulator of the high-temperature response in CYR34 [54]. Previous work has demonstrated that temperature reshapes transcriptional patterns in virulent CYR34-8 under elevated temperatures [40]. Our data show that temperature variation is associated with changes in the transcript abundance of genes annotated as molecular chaperones and metabolic proteins. These associations suggest—but do not establish—that protein homeostasis and metabolic adjustment may contribute to urediniospore germination across the tested temperature range.

**Figure 7:**
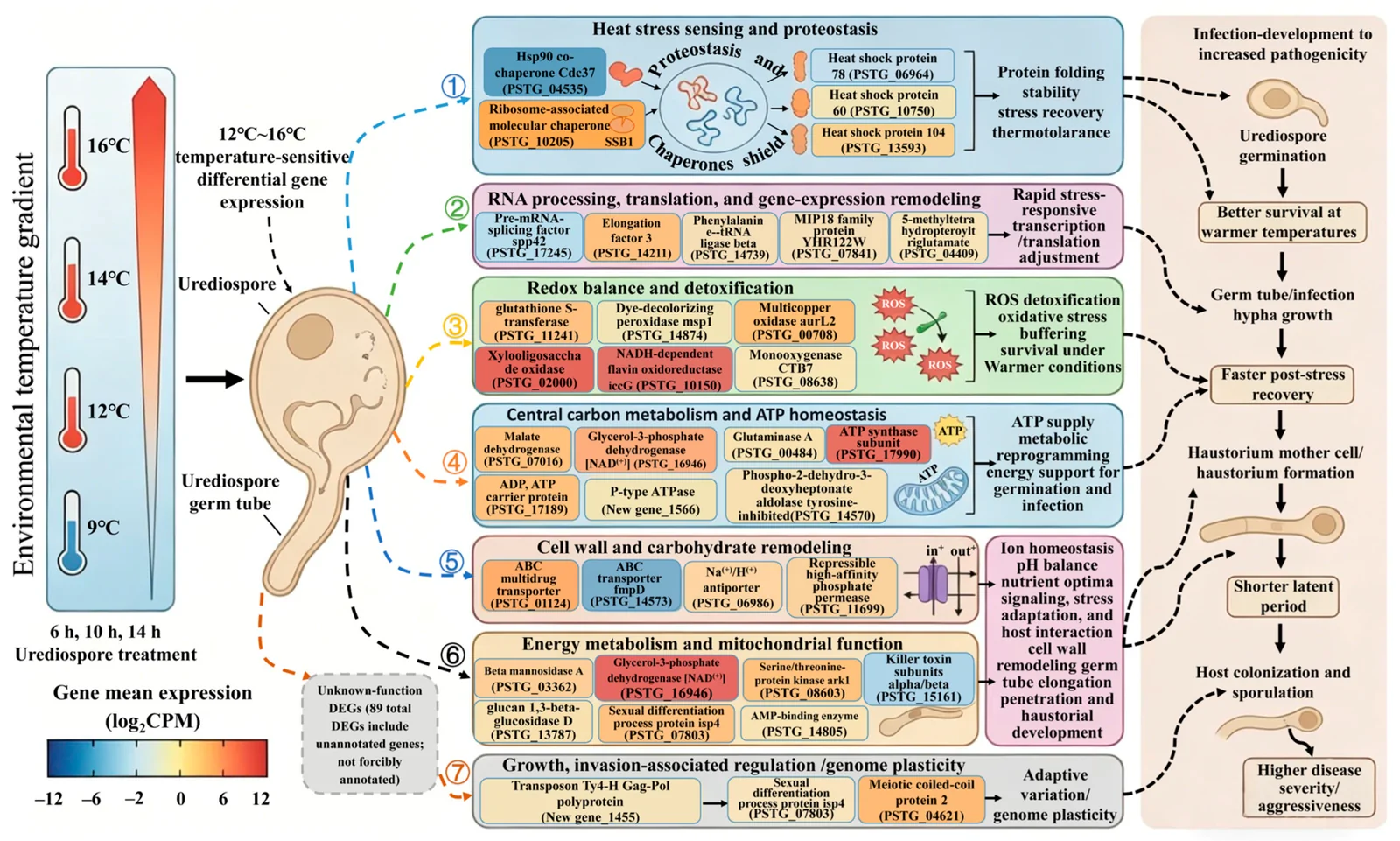
Proposed working model of biological processes potentially associated with temperature-responsive gene expression in germinating urediniospores of *Puccinia striiformis* f. sp. *tritici*. Left: Urediniospore treatments across 9–16 °C temperature gradient; Middle: Seven key functional pathways underlying heat adaptation, with major DEGs labelled in each cascade. color gradient represents gene expression levels. Solid arrows represent direct regulatory interactions, while dashed arrows represent indirect or putative regulatory relationships.

To systematically illustrate the molecular regulatory network underlying temperature sensitivity and pathogenicity in CYR34-8 urediniospores, we developed a hypothetical working model based on KEGG, GO, and PPI analysis. As shown in Figure 7, all 89 core DEGs were classified into seven interrelated functional modules according to their biological functions and expression patterns, collectively coordinating temperature adaptation and pathogenic variation in Pst race CYR34. Module 1 (heat stress sensing and proteostasis) and Module 2 (RNA processing factors) are predominantly activated at 12–16 °C. Multiple heat shock proteins and pre-mRNA splicing factors in these modules maintain protein stability and ensure proper transcription and translation regulation under elevated temperatures, serving as the first line of defense against thermal damage [55,65]. Module 3 (redox balance and oxidative detoxification) mainly functions at 12–14 °C, where oxidases and glutathione S-transferase scavenge ROS and alleviate oxidative stress [66,67]. Module 4 (central carbon metabolism and ATP homeostasis) is preferentially activated at 9 °C, providing sufficient ATP via carbon‑metabolism pathways for urediniospore germination, germ tube extension, and haustorium mother cell formation [68]. Module 5 (cell wall remodelling, ion and pH homeostasis) operates across the entire temperature range tested; ABC transporters and ion antiporters maintain intracellular homeostasis and nutrient uptake, thereby shortening the latent period of infection [69]. In addition, hydrolases and protein kinases in this module contribute to germ cell wall remodeling, germ tube elongation, and host tissue penetration. As an independent functional unit, Module 6 (energy metabolism and mitochondrial function) is preferentially activated at 9 °C, supplying ATP mainly derived from mitochondria to support key physiological processes, including urediniospore germination, germ tube growth, and haustorium mother cell formation [70]. Module 7 (growth, invasion-associated regulation, and genome plasticity) contains genes related to transposable elements and meiotic proteins, which may induce adaptive genomic variation and further enhance the pathogenic aggressiveness of Pst [71]. This proposed model summarizes the biological processes associated with the temperature-responsive expression profiles and provides testable hypotheses for future studies. The present results do not establish that elevated temperature sequentially activates these modules or that the identified genes directly enhance stress tolerance, metabolic fitness, or pathogenicity. These patterns suggest several processes that may be investigated as possible components of the temperature response in CYR34-8. However, the modules shown in Figure 7 should not be interpreted as experimentally established pathways or as evidence that the corresponding genes directly affect pathogenicity. In particular, ROS detoxification, ATP production, cell-wall remodeling, host penetration, infection latency, genomic variation, and pathogenic aggressiveness were not directly measured in the present study.

## 5. Conclusions

In conclusion, *Puccinia striiformis* f. sp. *tritici* (CYR34-8) exhibits significantly stronger thermal adaptability during urediniospore germination than CYR32-11 and CYR33-209. The identification of 89 shared temperature-responsive DEGs suggests that the response of CYR34-8 urediniospores to temperature variation may involve multiple genes and biological processes. Four genes predicted to encode an ABC transporter, malate dehydrogenase, a class 3 lipase, and Hsp70 showed relatively high connectivity in the predicted protein association network and were identified as candidates for future functional studies. Their annotations suggest possible associations with membrane transport, energy metabolism, lipid metabolism, and protein homeostasis, but their roles in thermal adaptation remain to be experimentally validated. Collectively, this study provides a transcriptomic resource and a set of candidate genes associated with the response of germinating CYR34-8 urediniospores to temperature variation. These results generate testable hypotheses for investigating thermal adaptation in *Pst* but do not establish causal molecular mechanisms or direct links between the identified genes and pathogen virulence.

## 6. Patents

Two Chinese utility model patents supporting the experimental procedures of this study have been authorized, as listed below:

1. Tao F, Wang C, Liu T, Zhou YH, Feng R. A wheat leaf dewaxing device. Chinese Utility Model Patent, Patent No. ZL 2021 2 2349939.7, Filing date: 27 September 2021, Grant publication date: 11 March 2022, Patentee: Gansu Agricultural University.
2. Tao F, Wang ZY, Wang LT, Shao X, Song MN. A portable spore collector. Chinese Utility Model Patent, Patent No. ZL 2024 2 0332619.1, Filing date: 22 February 2024, Grant publication date: 15 October 2024, Patentee: Gansu Agricultural University.

## Figures and Tables

**Table 1:** Significantly enriched KEGG pathways among 89 DEGs in *Puccinia striiformis* f. sp. *tritici* race CYR34-8.

Pathway	Pathway ID	*padj*	DEGs with Pathway Annotation	All Genes
Ubiquinone and other terpenoid-quinone biosynthesis	ko00130	0.001657	2 (*PSTG_00706, PSTG_14805*)	9
Other glycan degradation	ko00511	0.003528	2 (*PSTG_02000, PSTG_03362*)	13
Longevity regulating pathway	ko04213	0.004583	3 (*PSTG_06964, PSTG_10205, PSTG_13593*)	49
Legionellosis	ko05134	0.012886	2 (*PSTG_10205, PSTG_10750*)	25
ABC transporters	ko02010	0.0149469	2 (*PSTG_01124, PSTG_14573*)	27
Platinum drug resistance	ko01524	0.018286	2 (*PSTG_11241, PSTG_13751*)	30
Phenylpropanoid biosynthesis	ko00940	0.027790	1 (*PSTG_14805*)	4
Longevity regulating pathway-worm	ko04212	0.028554	2 (*PSTG_10750, PSTG_11241*)	38
Mineral absorption	ko04978	0.034621	1 (*PSTG_13751*)	5
Type I diabetes mellitus	ko04940	0.041405	1 (*PSTG_10750*)	6

*padj* represents adjusted *p*-value. “DEGs with pathway annotation” indicates the number and corresponding IDs of differentially expressed genes enriched in each pathway. “All genes” means the total number of all genes in the *Pst* genome annotated to the given KEGG pathway.

## Data Availability

All data generated or analyzed during this study are publicly available and freely downloadable, including the RNA-Seq dataset deposited in NCBI BioProject (https://www.ncbi.nlm.nih.gov/bioproject/PRJNA1289099 accessed on 9 July 2025) and the Supplementary Information Files attached to this manuscript.

## Supplementary Materials

The following supporting information can be downloaded at: https://www.mdpi.com/article/10.3390/jof12080582/s1. Figure S1: Principal component analysis (PCA) of gene expression profiles across all 36 RNA-Seq samples; Figure S2: KOG functional classification of shared temperature-responsive differentially expressed genes in *Puccinia striiformis* f. sp. *tritici* CYR34-8; Table S1: Primer of DEGs for qRT-PCR; Table S2: Q30 quality value of 36 RNA-Seq samples from CYR34-8; Table S3: Significantly enriched GO term 89 DEGs of CYR34-8; Supplementary File S1: SRA submission information for transcriptome sequencing samples of CYR34-8 under thermal gradient treatments; Supplementary File S2: FPKM of the 89 thermally responsive DEGs under gradient temperature treatments.

## Abbreviations

The following abbreviations are used in this manuscript:

Act	Actin
DEG	Differentially expressed gene
EF	Elongation factor
FPKM	Fragments Per Kilobase of transcript per Million mapped reads
GST	Glutathione S-transferase
Hsp	Heat shock protein
Log_2_FC	log_2_fold change
padj	Adjusted *p*-value
PHI	Pathogen-Host Interactions database
PPI	Protein–Protein Interaction
Pst	*Puccinia striiformis* f. sp. *tritici*
ROS	Reactive oxygen species
SRA	Sequence Read Archive
