# Supplementary material for: RNA-Seq Profiling Identifies Temperature-Responsive Genes in Germinating Urediniospores of Puccinia striiformis f. sp. tritici Race CYR34-8
Source: J Fungi (Basel). 2026 Aug 7;12(8):582. doi: 10.3390/jof12080582 (PMC13514815; doi:10.3390/jof12080582)
Supplement: Supplementary file 1 [file jof-12-00582-s001.zip › Tables S1-S3.pdf]

Table S1 Primer of DEGs for qRT-PCR

Gene ID	Primer sequence (5'～3')
PSTG_04409-F	5'ATGCCCTAATGTGACTGC 3'
PSTG_04409-R	5' AACAACTCTGATGGGTGATC 3'
PSTG_10150-F	5' ACGGTGCTGAAGTGCTTT 3'
PSTG_10150-R	5' TTGTTGACCCGAGGTGATA 3'
PSTG_06410-F	5' CCCTCTTAGTGTCCGTCAT 3'
PSTG_06410-R	5' CAAATAAGTTGCTTTCTCCC 3'
PSTG_02000-F	5' GTCGCCGATTCTGTCAAG 3'
PSTG_02000-R	5' GAGCAATGTCGCCAAGTC 3'
PSTG_04621-F	5' CAGAGCGAATAGGTGGTC 3'
PSTG_04621-R	5' GGTACTTAACGAGATGGTGC 3'
PSTG_07787-F	5' CCAGTTCAAGCCAGTTCG 3'
PSTG_07787-R	5' GATAGCATCGTGCCCAAA 3'
PSTG_00706-F	5' TCCCTTCATAGACCATACC 3'
PSTG_00706-R	5' GACTTCCATCTCCTCCTG 3'
PSTG_08976-F	5' GACGAAGCGGAGAACGAA 3'
PSTG_08976-R	5' GGCAAGCAATGAGGAGGA 3'
PSTG_14874-F	5' TCAAGTCCGGGATGTTAT 3'
PSTG_14874-R	5' GGGATGCGACGAGTGTAT 3'
PSTG_14374-F	5' ACGGATGTCGAATTTGCT 3'
PSTG_14374-R	5' GTCGGTGGTAAGGAGTGAA 3'
PSTG_06238-F	5' ACGCAGGCGGGTCACTAT 3'
PSTG_06238-R	5' TCCAAAGAACCAAAGGCACA 3'
PSTG_13751-F	5' GTATGTATGCGTTTAGGTTTC 3'
PSTG_13751-R	5' AATGATGGACGGATAGTGT 3
PSTG_14320-F	5' TTTTCGGGAAATCGTTAC 3'
PSTG_14320-R	5' AAGGTGCATCTTCTTTAGGT 3'

Table S2 Q30 quality value of 36 RNA-seq samples from CYR-34-8

Sample ID	Clean reads	Base Sum	GC%	Mapped reads	Q30%
C-9-6-1	16422792	4913724692	51.37	15584869	93.73
C-9-6-2	21662420	6484740270	52.91	18333574	94.15
C-9-6-3	15628472	4679476968	54.73	13663270	94.26
C-9-10-1	22037009	6593282898	53.69	17839136	94.17
C-9-10-2	25230849	7548172600	54.81	18779620	94.44
C-9-10-3	20272837	6063874276	53.02	17269747	93.98
C-9-14-1	20935353	6267113954	53.72	14332613	94.61
C-9-14-2	16740718	5015623874	54.02	12818247	93.72
C-9-14-3	19336548	5788876328	53.22	15346706	93.79
C-12-6-1	37305439	1117264924	50.97	32077664	94.53
C-12-6-2	16739570	5017718512	56.12	11573364	94.09
C-12-6-3	17999585	5392017172	56.06	13594401	94.26
C-12-10-1	22500708	6740828318	55.80	18111325	94.77
C-12-10-2	16057645	4813503768	55.09	12663528	93.6
C-12-10-3	19827437	5941115060	56.39	15023283	94.02
C-12-14-1	19229748	5762483506	55.31	12433605	94.49
C-12-14-2	27130866	8115310718	56.04	22285203	94.67
C-12-14-3	19657753	5886055166	55.52	15894268	94.53
C-14-6-1	19465727	5834567722	54.19	13785805	93.97
C-14-6-2	14256883	4272475434	53.98	10227857	93.88
C-14-6-3	21949096	6580322604	54.50	18841195	94.11
C-14-10-1	16090156	4822725448	51.00	12896099	92.23
C-14-10-2	17465450	5231755994	54.34	14234338	94.15
C-14-10-3	21075622	6316064204	54.30	18559994	94.26
C-14-14-1	16092924	4806834104	45.88	12197032	90.81
C-14-14-2	16197238	4851833782	50.48	12391118	93.02
C-14-14-3	16856705	5050593726	49.72	14211825	92.68
C-16-6-1	15216425	4560157306	50.19	10168781	92.95
C-16-6-2	16622331	4981227816	45.68	15789768	91.10
C-16-6-3	13792292	4132482216	49.99	10317685	92.92
C-16-10-1	19126515	5730865150	47.99	16529029	93.06
C-16-10-2	17171778	5144073764	47.77	12129806	91.70
C-16-10-3	16869688	5047125944	42.34	13554143	90.29
C-16-14-1	22353717	6695392268	41.41	19516317	89.45
C-16-14-2	23740338	7106744830	43.51	19676468	90.64
C-16-14-3	23748170	7103644780	42.22	18557350	90.09

Table S3 Significantly enriched GO term 89 DEGs of CYR-34-8

GO Term	Class	Descrption	*Padj*-value	DEGs_num	All genes_num
GO:0000712	Biological Process	resolution of meiotic recombination intermediates	0.006225	1	1
GO:0000735	Biological Process	removal of nonhomologous ends	0.006225	1	1
GO:0000736	Biological Process	double-strand break repair via single-strand annealing, removal of nonhomologous ends	0.006225	1	1
GO:0006277	Biological Process	DNA amplification	0.006225	1	1
GO:0006296	Biological Process	nucleotide-excision repair, DNA incision, 5'-to lesion	0.006225	1	1
GO:0006817	Biological Process	phosphate ion transport	0.006225	1	1
GO:0018131	Biological Process	oxazole or thiazole biosynthetic process	0.006225	1	1
GO:0033683	Biological Process	nucleotide-excision repair, DNA incision	0.006225	1	1
GO:0045002	Biological Process	double-strand break repair via single-strand annealing	0.006225	1	1
GO:0046484	Biological Process	oxazole or thiazole metabolic process	0.006225	1	1
GO:0051307	Biological Process	meiotic chromosome separation	0.006225	1	1
GO:0052837	Biological Process	thiazole biosynthetic process	0.006225	1	1
GO:0052838	Biological Process	thiazole metabolic process	0.006225	1	1
GO:1901255	Biological Process	nucleotide-excision repair involved in interstrand cross-link repair	0.006225	1	1
GO:0006790	Biological Process	sulfur compound metabolic process	0.010089	3	74

GO:0000710	Biological Process	meiotic mismatch repair	0.012413	1	2
GO:0000770	Biological Process	peptide pheromone export	0.012413	1	2
GO:0006108	Biological Process	malate metabolic process	0.012413	1	2
GO:0009914	Biological Process	hormone transport	0.012413	1	2
GO:0010817	Biological Process	regulation of hormone levels	0.012413	1	2
GO:0015698	Biological Process	inorganic anion transport	0.012413	1	2
GO:0006878	Biological Process	cellular copper ion homeostasis	0.018564	1	3
GO:0055070	Biological Process	copper ion homeostasis	0.018564	1	3
GO:0051604	Biological Process	protein maturation	0.019372	2	35
GO:0006624	Biological Process	vacuolar protein processing	0.024679	1	4
GO:0007534	Biological Process	gene conversion at mating-type locus	0.024679	1	4
GO:0035822	Biological Process	gene conversion	0.024679	1	4
GO:0044277	Biological Process	cell wall disassembly	0.024679	1	4
GO:0070871	Biological Process	cell wall organization involved in conjugation with cellular fusion	0.024679	1	4
GO:0071853	Biological Process	fungal-type cell wall disassembly	0.024679	1	4
GO:1904541	Biological Process	fungal-type cell wall disassembly involved in conjugation with cellular fusion	0.024679	1	4
GO:0044272	Biological Process	sulfur compound biosynthetic process	0.027313	2	42
GO:0005975	Biological Process	carbohydrate metabolic process	0.035312	4	206
GO:0036297	Biological Process	interstrand cross-link repair	0.042807	1	7
GO:0097428	Biological Process	protein maturation by iron-sulfur cluster transfer	0.048778	1	8
GO:0016021	Cellular Component	integral component of membrane	0.001057	11	670

GO:0031224	Cellular Component	intrinsic component of membrane	0.001146	11	676
GO:0016020	Cellular Component	membrane	0.002389	13	997
GO:0044425	Cellular Component	membrane part	0.006624	11	828
GO:0009331	Cellular Component	glycerol-3-phosphate dehydrogenase complex	0.013005	1	2
GO:0031520	Cellular Component	plasma membrane of cell tip	0.025849	1	4
GO:0045263	Cellular Component	proton-transporting ATP synthase complex, coupling factor F(o)	0.038535	1	6
GO:1990819	Cellular Component	actin fusion focus	0.038535	1	6
GO:0008901	Molecular Function	ferredoxin hydrogenase activity	0.005516	1	1
GO:0016695	Molecular Function	oxidoreductase activity, acting on hydrogen as donor	0.005516	1	1
GO:0016699	Molecular Function	oxidoreductase activity, acting on hydrogen as donor, iron-sulfur protein as acceptor	0.005516	1	1
GO:1990599	Molecular Function	3' overhang single-stranded DNA endodeoxyribonuclease activity	0.005516	1	1
GO:0000014	Molecular Function	single-stranded DNA endodeoxyribonuclease activity	0.011003	1	2
GO:0016615	Molecular Function	malate dehydrogenase activity	0.011003	1	2
GO:0030060	Molecular Function	L-malate dehydrogenase activity	0.011003	1	2
GO:0016491	Molecular Function	oxidoreductase activity	0.019925	4	195
GO:0004338	Molecular Function	glucan exo-1,3-beta-glucosidase activity	0.02189	1	4
GO:0005451	Molecular Function	monovalent cation:proton antiporter activity	0.02189	1	4

GO:0015081	Molecular Function	sodium ion transmembrane transporter activity	0.02189	1	4
GO:0015385	Molecular Function	sodium:proton antiporter activity	0.02189	1	4
GO:0061630	Molecular Function	ubiquitin protein ligase activity	0.0228	2	43
GO:0004553	Molecular Function	hydrolase activity, hydrolyzing O-glycosyl compounds	0.024833	2	45
GO:0061659	Molecular Function	ubiquitin-like protein ligase activity	0.025876	2	46
GO:0008422	Molecular Function	beta-glucosidase activity	0.027291	1	5
GO:0015297	Molecular Function	antiporter activity	0.027291	1	5
GO:0015298	Molecular Function	solute:cation antiporter activity	0.027291	1	5
GO:0015299	Molecular Function	solute:proton antiporter activity	0.027291	1	5
GO:0015491	Molecular Function	cation:cation antiporter activity	0.027291	1	5
GO:0099516	Molecular Function	ion antiporter activity	0.027291	1	5
GO:0016798	Molecular Function	hydrolase activity, acting on glycosyl bonds	0.030227	2	50
GO:0004826	Molecular Function	phenylalanine-tRNA ligase activity	0.032664	1	6
GO:0015926	Molecular Function	glucosidase activity	0.032664	1	6
GO:0003684	Molecular Function	damaged DNA binding	0.038008	1	7
GO:0015291	Molecular Function	secondary active transmembrane transporter activity	0.038008	1	7
GO:0022804	Molecular Function	active transmembrane transporter activity	0.039732	2	58
GO:0010181	Molecular Function	FMN binding	0.043324	1	8
GO:0004842	Molecular Function	ubiquitin-protein transferase activity	0.047515	2	64
GO:0016730	Molecular Function	oxidoreductase activity, acting on iron-sulfur proteins as donors	0.048613	1	9
